# Diagnostic performance of whole-body diffusion-weighted imaging for staging esophageal cancer: a comparative study with PET-CT

**DOI:** 10.2478/raon-2026-0037

**Published:** 2026-07-29

**Authors:** Xiaohu Xu, Rong Yu, Qiulian Ma, Tingting Yin, Haisheng Chen, Ye Qian, Hong Li, Rongxing Qi, Yachun Xu

**Affiliations:** 1 Department of Imaging, Haian People’s Hospital, Haian, Jiangsu Province, China; 2 Department of Thoracic Surgery, Haian People’s Hospital, Haian, Jiangsu Province, China; 3 Department of Oncology, Haian People’s Hospital, Haian, Jiangsu Province, China; 4 Department of Nuclear Medicine, Nantong First People’s Hospital Affiliated to Southeast University, Nantong, Jiangsu Province, China

**Keywords:** esophageal cancer, tumor staging, WB-DWI, PET-CT, diagnostic performance, comparative study

## Abstract

**Background:**

Accurate preoperative staging is crucial for selecting treatment strategies and assessing prognosis in esophageal cancer. Positron emission tomography-computed tomography (PET-CT) is currently recognized as an essential staging modality, whereas the value of whole-body diffusion-weighted imaging (WB-DWI), a radiation-free functional magnetic resonance imaging (MRI) technique in esophageal cancer staging remains unclear. The objective of the study was to conduct a head-to-head comparison of the diagnostic accuracy of WB-DWI versus PET-CT in the initial diagnosis of esophageal cancer patients for local T staging, regional lymph node (N) assessment, and distant metastasis (M) detection.

**Patients and methods:**

This study collected data from 96 patients with esophageal cancer who underwent pathological diagnosis and imaging evaluation at Haian People’s Hospital between January 2023 and January 2025. Propensity Score Matching (PSM) was employed to balance confounding factors between groups, ultimately forming matched cohorts for analysis: the WB-DWI group (n = 32) and the PET-CT group (n = 32). Using postoperative pathology (PTNM) and/or 6-month follow-up clinical diagnosis as the gold standard, the following metrics were evaluated: T staging accuracy, N staging sensitivity/specificity, distant metastasis (M staging) detection rate, overall staging accuracy, image signal-to-noise ratio (SNR), examination duration, and inter-observer agreement (Kappa value). Sensitivity, specificity, positive predictive value (PPV), negative predictive value (NPV), and accuracy were calculated for both methods. Diagnostic performance was analyzed using ROC curves, and the area under the curve (AUC) was compared via DeLong’s test.

**Results:**

WB-DWI and PET-CT demonstrated comparable overall staging accuracy for esophageal cancer (84.4% *vs*. 87.5%, *p* > 0.05). For N staging, PET-CT demonstrated higher sensitivity (90.6% *vs*. 78.1%, *p* < 0.05), while both methods showed identical specificity (87.5%). In detecting distant metastases, PET-CT exhibited marginally higher detection rates and accuracy than WB-DWI, though differences were not statistically significant. WB-DWI demonstrated lower accuracy for T staging (71.9% *vs*. 84.4%, p < 0.05). WB-DWI images exhibited significantly lower SNR than PET-CT (*p* < 0.01), but the examination was shorter in duration and showed good inter-observer agreement (Kappa > 0.60, *p* < 0.001).

**Conclusions:**

WB-DWI demonstrates comparable overall efficacy to PET-CT in esophageal cancer staging. Although its sensitivity and efficiency are slightly lower, its radiation-free nature and high specificity make it a viable supplement or alternative to PET-CT for specific patient populations. These findings provide evidence for precise clinical selection.

## Introduction

Esophageal cancer is one of the most prevalent malignant tumors globally, particularly in East Asia. Its insidious onset and aggressive nature often result in poor patient prognosis.^1,2^ Accurate preoperative staging is crucial for developing individualized treatment plans, assessing prognosis, and improving survival rates.^3,4^ Currently, clinical staging primarily relies on imaging examinations, aiming to precisely evaluate the depth of primary tumor invasion (T staging), regional lymph node metastasis (N staging), and the presence of distant metastasis (M staging).^5,6^ In this field, positron emission tomography-computed tomography (PET-CT), which integrates anatomical and functional information, is widely recognized as a vital staging tool.^7,8^ By reflecting glucose metabolic activity in tumor cells, PET-CT demonstrates high sensitivity in detecting lymph node and distant metastases, significantly influencing treatment strategy adjustments.^9,10^ However, this technology also has inherent limitations, including relatively high examination costs, exposure to ionizing radiation, and the potential for false-positive results in certain inflammatory or physiological uptake scenarios. These factors have somewhat constrained its routine and repetitive clinical application.^11,12^

With the rapid advancement of magnetic resonance imaging (MRI) technology, whole-body diffusion-weighted imaging (WB-DWI) has gained increasing attention in oncological assessment as a radiation-free functional imaging modality.^13,14^ This technique reflects cellular density by detecting the degree of restriction to water-molecule diffusion within tissues, with malignant tissues typically exhibiting higher signal intensity.^15,16^ Theoretically, WB-DWI holds the potential to perform whole-body tumor screening and staging in a single examination while avoiding radiation exposure.^17,18^ Preliminary studies in recent years have explored the value of WB-DWI in evaluating systemic tumors such as breast cancer and lymphoma. However, systematic evidence supporting its application in staging esophageal cancer, a malignancy with unique anatomical and biological characteristics, remains lacking.^19,20^ The existing literature primarily consists of small-sample exploratory studies or those that have not conducted rigorous, methodologically robust direct comparisons with the current gold standard, PET-CT. Therefore, clarifying the specific diagnostic efficacy of WB-DWI across all tumor node metastasis (TNM) stages of esophageal cancer and delineating its advantages and limitations has clear practical significance for enriching clinical imaging options and optimizing diagnostic and therapeutic pathways.

Against this background, this study aims to systematically evaluate the diagnostic performance of WB-DWI in staging newly diagnosed esophageal cancer patients through a retrospective, head-to-head comparative analysis, using PET-CT as the reference standard. The study addresses several key questions: Is there a difference between WB-DWI and PET-CT in overall TNM staging accuracy? How do the sensitivity and specificity of the two imaging techniques perform in specific tasks such as assessing local invasion for T staging, lymph node metastasis for N staging, and distant metastasis for M staging? Beyond diagnostic efficacy, operational metrics such as examination duration, image quality, and consistency in result interpretation are equally important dimensions in clinical practice. Addressing these questions will contribute to a more comprehensive understanding of WB-DWI’s clinical applicability.

The innovation of this study lies in its adoption of a more rigorous research design to enhance the reliability of its conclusions. We collected consecutive case records from Hai’an People’s Hospital over the past two years. All enrolled patients received pathological confirmation and underwent comprehensive imaging evaluations. To control potential confounding factors affecting comparative outcomes, this study employed Propensity Score Matching (PSM) to balance baseline clinical characteristics between the WB-DWI and PET-CT assessment groups. This approach enabled the construction of a more comparable matched cohort for analysis. For evaluation metrics, we established multidimensional assessment criteria encompassing not only core diagnostic indicators, such as accuracy, sensitivity, and specificity for traditional T, N, M staging, but also parameters reflecting technical utility and stability, such as image signal-to-noise ratio (SNR), examination duration, and inter-observer agreement. Through this comprehensive comparison, we aim not only to validate WB-DWI’s fundamental diagnostic capabilities but also to provide more detailed evidence from a clinical practice perspective for its positioning, whether as an effective alternative in specific scenarios (*e.g*., iodine contrast agent contraindications, radiation avoidance requirements) or as a complementary diagnostic tool to PET-CT. The findings of this study are expected to provide clinically relevant, evidence-based insights from Chinese population data to develop personalized imaging staging protocols for esophageal cancer.

## Patients and methods

### Research subjects

**FIGURE 1.**
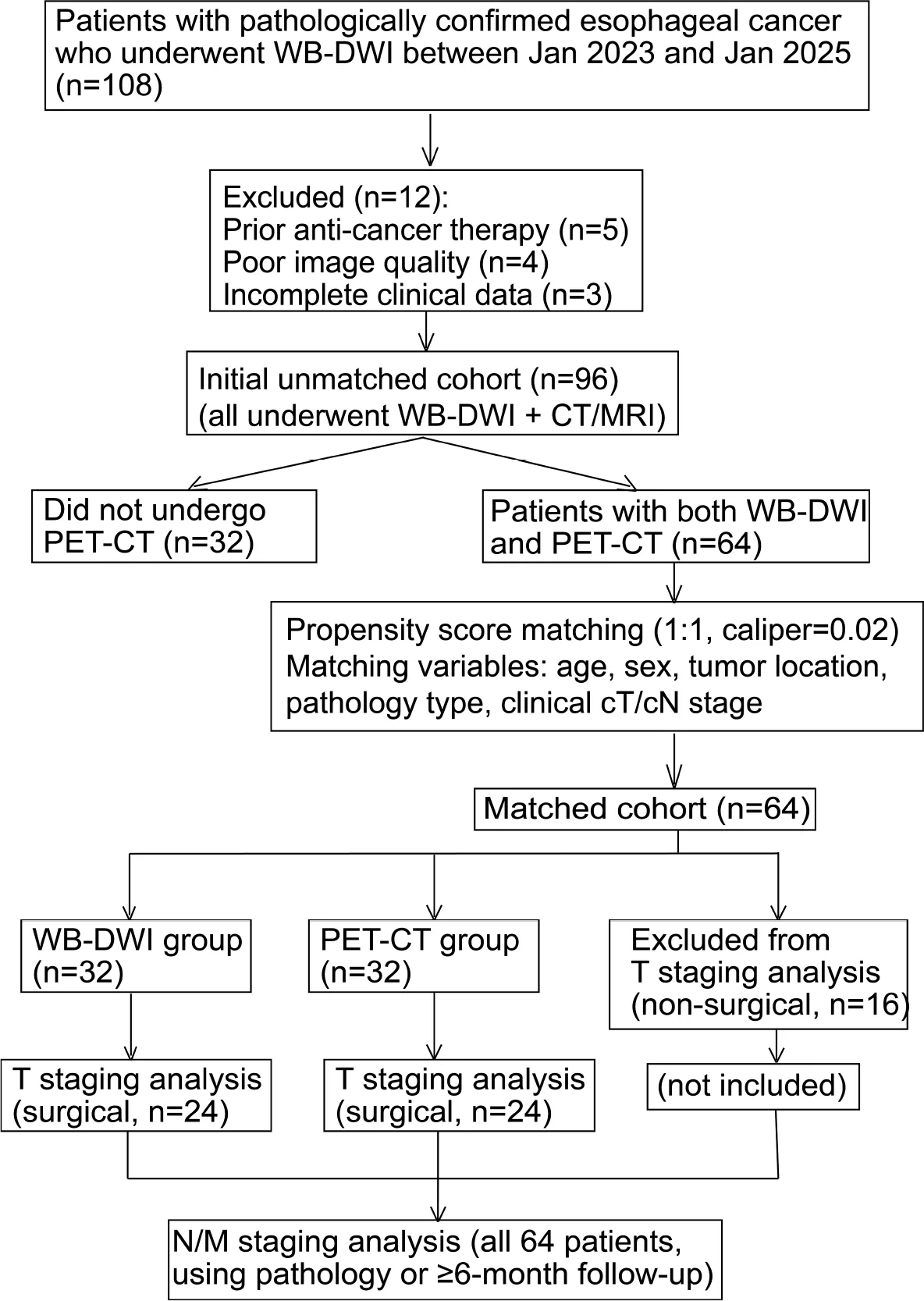
Flowchart. WB-DWI = whole-body diffusion-weighted imaging

This study employed a retrospective cohort design. The subjects were esophageal cancer patients who sought treatment at Hai’an People’s Hospital between January 2023 and January 2025. Initially, 108 newly diagnosed patients with pathologically confirmed diseases and complete imaging data were identified. By strictly applying inclusion and exclusion criteria, we established an initial study cohort of 96 patients. All patients underwent WB-DWI and completed one or more conventional CT and/or MRI examinations within a 2-week interval for clinical staging. Among these, 64 patients also underwent 18F-FDG PET-CT examinations concurrently. To fairly compare the diagnostic performance of the two imaging modalities and to control for selection bias and confounding factors, we performed propensity score-matching analysis on the 64 patients with both WB-DWI and PET-CT data. Matching variables included key clinical and pathological factors potentially affecting staging accuracy: age, sex, primary tumor location (cervical, thoracic, abdominal), histological type (squamous cell carcinoma, adenocarcinoma), and preliminary clinical staging (based on conventional imaging). Through 1:1 nearest-neighbor matching with a caliper value of 0.02, a matched cohort of 32 patient pairs was successfully established, comprising a WB-DWI evaluation group and a PET-CT evaluation group, each containing 32 cases. The matched groups demonstrated good balance in the matched variables, as shown in Figure 1.

### Inclusion and exclusion criteria

*Inclusion criteria*:^21^ (1) The biopsy under gastroscopy confirmed it to be esophageal cancer (squamous cell carcinoma or adenocarcinoma), and a complete pathological report was available.; (2) Newly diagnosed and not previously treated with any anticancer therapy; (3) Completed chest and whole-abdominal contrast-enhanced CT, wholebody WB-DWI, and whole-body PET-CT examinations within the same treatment period (interval ≤ 4 weeks); (4) Possesses complete basic clinical information.

*Exclusion criteria*:^22^ (1) History of other active malignancies; Poor image quality with severe artifacts significantly impairing interpretation; Severe deficiencies in clinical or follow-up data preventing determination of final clinical staging; Examination intervals exceeding the specified time window.

## Research plan

The PET-CT examination was conducted using the Biograph mCT 64-slice scanner produced by Siemens of Germany. Before the examination, patients were required to fast for more than 6 hours, and their blood sugar levels were controlled within the normal range. They were intravenously injected with ^18^F-FDG tracer (3.7–4.4 MBq/kg) based on their weight, and the scan was performed 60 minutes after rest. The scanning range extended from the top of the skull to the middle of the thigh. Unlike conventional PET/CT, the PET/CT examination in this study also included diagnostic contrast-enhanced CT: after injecting the iodine contrast agent (iodiprone, 1.5 mL/kg, injection rate 3 mL/s), images were collected respectively during the arterial phase and the venous phase, with a layer thickness of 1.25 mm and a reconstruction interval of 1.0 mm. This diagnostic CT was used to precisely assess the T stage of the primary tumor (the depth of esophageal wall infiltration, the relationship with the surrounding fat space and adjacent organs) and the morphological characteristics of lymph nodes. The PET data was corrected for attenuation using low-dose CT and fused with the diagnostic CT images.^23,24^

WB-DWI examinations were performed on the Ingenia CX 3.0T MRI system (Philips Healthcare, Netherlands). Patients are positioned supine with a body phased-array coil. The scanning sequence is a single-shot planar echo-planar imaging (EPI) sequence. The scan range covers from the cranial vault to the mid-thigh. Key parameters are as follows: TR/TE 5600/70 ms, field of view (FOV) 380×380 mm, matrix 128×128, slice thickness 6 mm, slice spacing 1 mm. DWI utilized three b-values (0, 800, 1000 s/mm^2^). The system automatically generated diffusion-weighted images at a high b-value (b = 1000 s/mm^2^) and corresponding apparent diffusion coefficient maps.^25,26^

Regarding imaging interpretation standards, all images were independently analyzed by two senior radiologists under double-blind conditions. Staging was strictly according to the 8^th^ edition of the American Joint Committee on Cancer (AJCC) TNM classification for esophageal and gastroesophageal junction cancers.^27^ For PET-CT images, the criterion for determining positive lymph node metastasis is as follows: the maximum standardized uptake value (SUVmax) of the lymph node exceeds 2.5 times the SUVmax of the mediastinal blood pool, or exceeds the background SUVmax of the liver. That is: lymph node SUVmax > 2.5 × SUVmax (mediastinal blood pool) or lymph node SUVmax > SUVmax (liver background). For WB-DWI images, lymph nodes exhibiting marked hyperintensity on high b-value images and hypointensity on ADC maps were considered suspicious for metastasis. For each suspicious lymph node detected by WB-DWI and PET-CT, two physicians jointly measured its short diameter (taking the maximum diameter perpendicular to the long axis on the cross-sectional image), and recorded the measurement in the structured report. For metastatic lymph nodes confirmed by pathology or follow-up, the minimum short diameter value was further recorded to evaluate the detection lower limit of the two imaging techniques for small lymph node metastasis. The determination of T stage of the primary lesion mainly relies on the anatomical morphological features of diagnostic enhanced CT (or MRI), including the degree of esophageal wall thickening, whether the layers of the wall are clear, whether the periphery is smooth, whether there are cord-like shadows or blurring in the surrounding fat space, and whether there is direct invasion of adjacent organs (trachea, aorta, pericardium, vertebrae). At the same time, the abnormal distribution range and pattern of metabolic abnormalities of PET (such as whether FDG uptake exceeds the esophageal contour) serve as supplementary reference information.^28^ In cases of diagnostic disagreement, a third senior expert reviewed the findings to reach consensus.

### Observation indicators

This study established comprehensive and multilevel evaluation criteria to systematically compare the performance of WB-DWI and PET-CT in esophageal cancer staging across three dimensions: diagnostic accuracy, image quality, and clinical utility. All the T-stage determinations based on imaging examinations were based solely on the postoperative pathological examination (pTNM) results as the gold standard. Therefore, the accuracy analysis of T-stage was limited to patients who underwent radical surgery and received complete pathological specimens. The T-stage of patients who did not undergo surgery was not included in the accuracy analysis. For N-stage and M-stage, the gold standard was defined as follows: For patients who undergo radical surgery and lymph node dissection, the postoperative pathological report shall be taken as the standard; for patients who have not undergone surgery or whose lymph nodes have not been dissected, if they undergo endoscopic ultrasound-guided fine-needle aspiration (EUS-FNA) and obtain a positive pathological result, it is determined as metastasis; if there is no pathological result, the reference standard is the comprehensive judgment based on at least 6 months of clinical follow-up (including subsequent progressive enlargement of lymph nodes on imaging or shrinkage after treatment for the metastatic lesion).

The specific detection indicators and their judgment criteria are as follows: (1) For primary tumors, evaluate the diagnostic accuracy of T staging, specifically the proportion of cases where imaging T staging and pathological T staging are completely consistent.^29^ (2) For regional lymph node metastasis, evaluate the diagnostic performance of N staging by calculating its sensitivity (true positive rate), specificity (true negative rate), positive predictive value (PPV), and negative predictive value (NPV). The determination of lymph node metastasis status shall be based on pathological results or comprehensive follow-up diagnosis. (3) For distant metastasis, evaluate the diagnostic performance of M staging by calculating its detection rate, sensitivity, specificity, and accuracy, with the gold standard being distant metastatic lesions confirmed by pathology or follow-up. (4) Assess the overall accuracy of TNM staging, defined as the proportion of cases where imaging-based staging perfectly aligns with the final pathologically confirmed clinical stage in stages I, II, III, and IV.^30^

In addition to the aforementioned core diagnostic indicators, this study introduced an image quality assessment system combining objective and subjective measures. The objective metric was the image SNR, calculated by the operating engineer after measuring the target lesion signal intensity and background noise standard deviation within a fixed region of interest. Subjective evaluation used a specially designed 5-point Likert scale, with two radiologists independently scoring the overall image quality for each examination. The scoring criteria were as follows: 1 point (Poor image quality, extreme noise, unidentifiable anatomical structures, unusable for diagnosis); 2 points (Fairly poor image quality, noticeable noise, blurred anatomical structures, low diagnostic confidence); 3 points (Average image quality, some noise present, but key anatomical structures still recognizable, basic diagnosis possible); 4 points (good image quality, well-controlled noise, clear anatomical structures, high diagnostic confidence); 5 points (excellent image quality, minimal noise, sharp and clear anatomical details, ideal diagnostic image).^31^ The two physicians’ scores were used to calculate inter-observer agreement, with the average score serving as the final subjective image quality score for each case.

Additionally, this study will document and compare the examination duration for both techniques, defined as the total time from patient entry into the examination room until completion of all image acquisition and exit from the room. Finally, an integrated assessment of interobserver agreement will be conducted, encompassing not only Kappa consistency analysis for T, N, M, and overall staging determinations, but also intraclass correlation coefficient (ICC) analysis for the aforementioned subjective image quality ratings. All statistical analyses will strictly adhere to the predefined protocol to ensure the scientific rigor of comparisons and the reliability of conclusions.

### Evaluation process for distant metastases

In this study, the assessment of distant metastatic lesions follows a tiered, gold-standard process anchored by objective evidence independent of the imaging studies being evaluated. The specific criteria are as follows:

First, at the imaging interpretation level, the evaluating physician documents all suspected distant metastatic lesions based on WB-DWI or PET-CT imaging findings, such as abnormal discoveries in the liver, lungs, bones, adrenal glands, or non-regional lymph nodes. On WB-DWI, metastatic lesions typically appear markedly hyperintense on high b-value images and hypodense on corresponding apparent diffusion coefficient maps. On PET-CT, metastatic lesions appear as focal areas of abnormal fluorodeoxyglucose (FDG) metabolism inconsistent with physiological uptake at corresponding anatomical locations.

However, imaging abnormalities alone do not constitute a definitive diagnosis. The gold standard for confirming distant metastasis in this study is established by high-level evidence in the following order of priority: 1. Pathological confirmation: Tissue obtained via needle biopsy or surgical resection, pathologically confirmed as esophageal cancer metastasis, represents the highest level of evidence.^32^ 2. Follow-up imaging confirmation: For lesions without pathological confirmation, subsequent imaging studies (*e.g*., contrast-enhanced CT, contrast-enhanced MRI, or follow-up PET-CT) performed at least 3 months later must demonstrate clear features of malignant progression, such as progressive enlargement, increased number, or emergence of new typical malignant signs (*e.g*., osteolytic lesions caused by bone metastasis).^33^ 3. Confirmation by clinical treatment response: During systemic antitumor therapy (*e.g*., chemotherapy, targeted therapy, or immunotherapy), the lesion significantly shrinks or disappears in response to effective treatment, and enlarges or recurs when treatment fails or progresses.^34^

#### Comprehensive Evaluation Process

The study will integrate all available information. For a suspected lesion, if confirmed by either criterion 1 or 2 above, it is definitively diagnosed as a metastatic lesion. If criterion 3 is met and consensus is reached during multidisciplinary consultation, it is also deemed metastatic. Conversely, if an imaging-suspicious lesion remains stable or shows no malignant progression during long-term followup (≥ 6 months) and cannot be explained by other benign lesions, it should remain under suspicion and be cautiously excluded. If follow-up confirms benignity (*e.g*., cyst, hemangioma) or if the lesion remains stable long-term and is determined benign by multidisciplinary discussion, it is classified as negative. The final M stage classification for all cases will be adjudicated using this comprehensive gold standard, which will serve as the basis for evaluating the diagnostic performance of WB-DWI and PET-CT at initial diagnosis.

### Sample size calculation

Given the retrospective nature of this study, the sample size was not determined prospectively but rather based on the available eligible consecutive cases in our hospital’s imaging database during the study period. We systematically collected all newly diagnosed esophageal cancer patients with pathologically confirmed diagnoses who underwent both WB-DWI and PET-CT examinations between January 2023 and January 2025, resulting in an initial cohort of 96 cases. To control baseline confounding factors, cases were screened and matched using propensity score matching, yielding a matched analysis set comprising 64 patients (32 in the WB-DWI group and 32 in the PET-CT comparison group). A post-hoc power analysis was conducted. Based on Weijun Zhao *et al.’s* reported overall staging accuracy (P1) of approximately 85% for WB-DWI and 90% (P2) for PET-CT.^35^ Retrospective calculations were performed using the paired proportion comparison formula at α = 0.05 (two-tailed). Analysis indicates that the current matched sample size provides approximately 80% statistical power to detect the observed effect difference, confirming its adequacy for the comparative analysis.

### Statistical methods

Statistical analysis methods will be rigorous and comprehensive. Quantitative data will be expressed as mean ± standard deviation or median (interquartile range), and intergroup comparisons will be performed using independent-samples t-tests or Mann-Whitney U tests. Qualitative data will be presented as frequencies (percentages), and intergroup comparisons will be conducted using chi-square tests or Fisher’s exact test. Postoperative pathological staging (PTNM) and/or comprehensive clinical follow-up results for at least 6 months (for non-surgical patients) will serve as the diagnostic gold standard. Sensitivity, specificity, PPV, NPV, and accuracy for each staging indicator will be calculated separately for WB-DWI and PET-CT. Receiver operating characteristic (ROC) curve analysis was used to evaluate the diagnostic performance of both methods for N and M staging, with differences in area under the curve (AUC) assessed via DeLong’s test. Inter-observer agreement was evaluated using the Kappa coefficient. All statistical analyses were performed using SPSS 26.0, with *p* < 0.05 considered statistically significant.

After propensity score matching, two independent and baseline-balanced groups were formed: the WB-DWI assessment group (n = 32) and the PET-CT assessment group (n = 32). For each diagnostic performance indicator (T stage accuracy, N stage sensitivity/specificity, M stage diagnostic efficacy, overall TNM accuracy), it was independently calculated based on the patients in each group and their corresponding gold standards. For example, the N stage sensitivity of WB-DWI was derived from the comparison of the WB-DWI interpretation results of the 32 patients in this group with the gold standard; the N stage sensitivity of PET-CT was derived from the comparison of the PET-CT interpretation results of the 32 patients in this group with the gold standard. Therefore, in the result table, each technique presents a set of diagnostic parameters, and the total number of diagnostic results is 64 (32 + 32). In Tables 2 to 5, “n = 64” indicates the total of the diagnostic results of the two techniques, rather than calculating a single indicator by mixing the data from the two groups.

### Ethics statement

This study strictly adheres to medical ethics standards. The research protocol has been submitted to and approved by the Ethics Review Committee of Hai’an People’s Hospital. Given that this study is a retrospective analysis involving no additional invasive procedures, and all data will be analyzed after de-identification, the Ethics Committee has approved an exemption from obtaining patient-informed consent. Throughout the study, patient privacy and data security will be rigorously protected, and all information will be used solely for this research.

## Results

### Baseline information

Before PSM, there were noticeable differences in several baseline variables between the two groups, particularly in age (SMD = 0.38) and tumor length (SMD = 0.29). After 1:1 nearest-neighbor matching with a caliper of 0.02, all variables achieved SMD <0.1, indicating successful balance (Table 1A and 1B). After propensity score matching (Table 1B), 32 patients were enrolled in each of the WB-DWI and PET-CT groups. As shown in Table 1, no statistically significant differences were observed between the two groups in all 11 baseline characteristics, including age, gender, tumor location, histological type, clinical preliminary staging (cT, cN), tumor length, differentiation grade, macroscopic subtype, clinical symptoms, and body mass index (*p* > 0.05). This indicates successful matching and good comparability between groups.

**TABLE 1A.** Baseline characteristics before propensity score matching (unmatched cohort, n = 64)

Variable	WB-DWI group (n = 32)	PET-CT group (n = 32)	SMD
**Age (years), mean ± SD**	62.5 ± 9.1	65.8 ± 8.2	0.38
**Gender, male, n (%)**	23 (71.9)	25 (78.1)	0.14
**Tumor location, n (%)**			
**Cervical**	2 (6.3)	3 (9.4)	0.12
**Thoracic**	24 (75.0)	23 (71.9)	0.07
**Abdominal**	6 (18.7)	6 (18.7)	0.00
**Pathological type, n (%)**			
**Squamous cell carcinoma**	27 (84.4)	26 (81.3)	0.08
**Adenocarcinoma**	5 (15.6)	6 (18.7)	0.08
**Preliminary clinical T stage, n (%)**			
**cT1-2**	13 (40.6)	15 (46.9)	0.13
**cT3-4**	19 (59.4)	17 (53.1)	0.13
**Preliminary clinical N stage, n (%)**			
**cN0**	10 (31.3)	12 (37.5)	0.13
**cN+**	22 (68.7)	20 (62.5)	0.13
**Tumor length (cm), mean ± SD**	4.3 ± 1.6	4.9 ± 1.8	0.35
**Degree of differentiation, n (%)**			
**High**	4 (12.5)	5 (15.6)	0.09
**Medium**	18 (56.3)	19 (59.4)	0.06
**Low**	10 (31.3)	8 (25.0)	0.14
**Body mass index (kg/m^2^), mean ± SD**	21.9 ± 3.0	22.3 ± 2.9	0.14

1 SMD = standardized mean difference; WB-DWI = whole-body diffusion-weighted imaging

1 SMD > 0.3 indicates moderate to large imbalance. Before matching, notable imbalances were observed in age (SMD = 0.38) and tumor length (SMD = 0.35).

**TABLE 1B.** Baseline characteristics after propensity score matching (matched cohort, n = 64)

Variable	WB-DWI group (n = 32)	PET-CT group (n = 32)	SMD
**Age (years), mean ± SD**	63.2 ± 8.5	64.1 ± 7.9	0.11
**Gender, male, n (%)**	24 (75.0)	25 (78.1)	0.07
**Tumor location, n (%)**			
**Cervical**	2 (6.3)	3 (9.4)	0.12
**Thoracic**	25 (78.1)	23 (71.9)	0.14
**Abdominal**	5 (15.6)	6 (18.7)	0.08
**Pathological type, n (%)**			
**Squamous cell carcinoma**	28 (87.5)	26 (81.3)	0.17
**Adenocarcinoma**	4 (12.5)	6 (18.7)	0.17
**Preliminary clinical T stage, n (%)**			
**cT1-2**	14 (43.8)	13 (40.6)	0.06
**cT3-4**	18 (56.2)	19 (59.4)	0.06
**Preliminary clinical N stage, n (%)**			
**cN0**	11 (34.4)	10 (31.3)	0.07
**cN+**	21 (65.6)	22 (68.7)	0.07
**Tumor length (cm), mean ± SD**	4.5 ± 1.8	4.7 ± 1.6	0.12
**Degree of differentiation, n (%)**			
**High**	5 (15.6)	4 (12.5)	0.09
**Medium**	18 (56.3)	20 (62.5)	0.13
**Low**	9 (28.1)	8 (25.0)	0.07
**Body mass index (kg/m^2^) mean ± SD**	21.8 ± 3.2	22.2 ± 2.9	0.13

1 SMD = standardized mean difference; WB-DWI = whole-body diffusion-weighted imaging

1 After 1:1 nearest-neighbor matching with a caliper of 0.02, all variables achieved SMD < 0.2, with most < 0.1, indicating successful balance between the two groups. The matched cohort consisted of 32 patients per group (total n = 64).

### Primary diagnostic performance indicators

Among the 64 matched patients after PSM, a total of 48 cases (75.0%) underwent radical esophageal cancer resection and achieved complete pathological T staging. Among them, 24 cases were in the WB-DWI group and 24 cases were in the PET-CT group. The remaining 16 patients did not undergo surgery due to distant metastasis (n = 10), locally advanced inoperability (n = 4), or poor general condition making them intolerant to surgery (n = 2). Their T staging was not included in the accuracy analysis. Therefore, the T staging accuracy rate in Table 2 was calculated based on 48 surgical patients (24 cases in each group). Regarding primary diagnostic efficacy indicators, the two imaging modalities exhibit distinct characteristics. The following diagnostic performance indicators were independently calculated for each of the two groups of patients (32 cases in each group) after matching. To facilitate presentation, the results of both techniques are listed in the table, and the total number of diagnostic cases is indicated as 64. As shown in Table 2, PET-CT demonstrated a trend toward higher accuracy (84.4%) in primary tumor T staging compared with WB-DWI (71.9%), though the difference was not statistically significant (*p* > 0.05). For regional lymph node (N staging) assessment, Table 3 data indicate that PET-CT significantly outperformed WB-DWI in sensitivity (90.6% *vs*. 78.1%, *p* < 0.05), while both techniques demonstrated comparable specificity (87.5% each). Regarding the detection of distant metastasis (M staging), Table 4 shows that both techniques performed well. PET-CT showed marginally higher values for sensitivity (91.7% *vs*. 83.3%) and specificity (94.2% *vs*. 92.3%), but the differences were not statistically significant. Regarding overall staging capability, Table 5 shows that WB-DWI and PET-CT demonstrated very similar overall TNM staging accuracy rates of 84.4% and 87.5%, respectively, with no statistically significant difference between them (*p* > 0.05).

**TABLE 2.** Comparison of diagnostic accuracy in T staging (n = 32, compared with pathology results)

Imaging technology	Number of accurate diagnoses	Diagnostic accuracy (%)
**WB-DWI**	23	71.9
**PET-CT**	27	84.4
χ^2^ = 1.60, *p* = 0.206		

1 WB-DWI = whole-body diffusion-weighted imaging

1 Accurate diagnosis refers to complete concordance between imaging T staging and pathological pT staging.

**TABLE 3.** Comparative diagnostic performance of N staging (n = 64, all lymph node regions)

Imaging technology	Sensitivity (%)	Specificity (%)	PPV (%)	NPV (%)	Accuracy rate (%)
**WB-DWI**	78.1 (25/32)	87.5 (28/32)	86.2	80.0	82.8
**PET-CT**	90.6 (29/32)	87.5 (28/32)	87.9	90.3	89.1

1 PPV = positive predictive value; NPV = negative predictive value; WB-DWI = whole-body diffusion-weighted imaging

1 Numbers in parentheses indicate sample size. Sensitivity comparison: c^2^ = 4.01, *p* = 0.045. In this table, the sensitivity of WB-DWI (78.1%, 25/32) is calculated based on the 32 patients in the WB-DWI group; the sensitivity of PET-CT (90.6%, 29/32) is calculated based on the 32 patients in the PET-CT group. The notation “n = 64” at the top of the table merely indicates that there are a total of 64 diagnostic results from both techniques (32 + 32), and it does not mean that the two groups of data are combined.

**TABLE 4.** Comparative diagnostic performance of M staging (n = 64, patient level)

Imaging Technology	Sensitivity (%)	Specificity (%)	PPV (%)	NPV (%)	Accuracy rate (%)
**WB-DWI**	83.3 (10/12)	92.3 (48/52)	76.9	94.1	90.6
**PET-CT**	91.7 (11/12)	94.2 (49/52)	84.6	96.1	93.8

1 PPV = positive predictive value; NPV = negative predictive value; WB-DWI = whole-body diffusion-weighted imaging

1 1. The gold standard is pathological confirmation or confirmation via follow-up. The diagnostic parameters for WB-DWI in the table are calculated based on the 32 patients in the WB-DWI group; the diagnostic parameters for PET-CT are calculated based on the 32 patients in the PET-CT group. The notation “n = 32” at the top of the table corresponds to the sample size of each group and does not indicate that data from the two groups have been combined.

1 2. Sensitivity comparisons were performed using Fisher’s exact test, with *p* = 1.000.

1 3. Explanation of the denominators 12 and 52: Among the 32 patients in the WB-DWI group, 6 were gold standard positive (M1) and 26 were negative (M0); in the PET-CT group of 32 patients, 6 were gold standard positive (M1) and 26 were negative (M0). In the table, 10/12 represents 10 true positives in the WB-DWI group (out of a total of 12 actual positives, *i.e*., the sum of positives from both groups), and 48/52 represents the sum of true negatives from both groups. To avoid misunderstanding, we clarify: the “12” in 10/12 refers to the total number of positive cases across both groups, and the “52” in 48/52 refers to the total number of negative cases across both groups. The sensitivity and specificity for both techniques were calculated based on data within their respective groups and then presented as aggregated results.

**TABLE 5.** Comparison of overall accuracy in TNM staging (n = 64)

Imaging technology	Number of accurate diagnoses	Overall staging accuracy rate (%)
**WB-DWI**	54	84.4
**PET-CT**	56	87.5

1 WB-DWI = whole-body diffusion-weighted imaging

1 Accurate diagnosis refers to complete agreement between the overall radiological staging and the final pathological/clinical staging (Stages I, II, III, or IV). 2. The accuracy rate for the WB-DWI group is calculated based on the 32 patients in that group; the accuracy rate for the PET-CT group is calculated based on the 32 patients in that group. The “n = 64” above the table represents the total number of diagnostic results for both techniques (32+32) and does not indicate a single accuracy rate calculated by combining data from both groups. 3. Intergroup comparisons were performed using the chi-square test: χ^2^ = 0.22, *p* = 0.639.

### Secondary technical and practical performance metrics

The contrast between the two modalities is even more pronounced in secondary technical and practical metrics. As shown in Table 6, PET-CT significantly outperformed WB-DWI in both objective image quality measures (SNR) and subjective ratings (5-point scale) (*p* < 0.001). However, WB-DWI demonstrated a clear advantage in efficiency, with a significantly shorter average scan duration than PET-CT (28.5 minutes *vs*. 45.3 minutes, *p* < 0.001).

**TABLE 6.** Objective and subjective evaluation of image quality and examination duration

Evaluation Indicators	WB-DWI group (n = 32)	PET-CT group (n = 32)	F	*p*
**Image signal-to-noise ratio, (mean ± SD)**	15.2 ± 4.1	22.8 ± 5.6	t = 6.42	< 0.001
**Subjective quality rating (5-point scale, mean ± SD)**	3.8 ± 0.7	4.5 ± 0.5	t = 4.76	< 0.001
**Inspection time (minute, mean ± SD)**	28.5 ± 5.2	45.3 ± 8.7	t = 9.83	< 0.001

1 WB-DWI = whole-body diffusion-weighted imaging

The results of the interobserver agreement analysis (Table 7) indicate that both radiologists demonstrated good to excellent agreement when using the two staging techniques (all Kappa values > 0.60, *p* < 0.001). Specifically, the consistency coefficients for PET-CT (Kappa values ranging from 0.78 to 0.85) were generally slightly higher than those for WB-DWI (Kappa values ranging from 0.65 to 0.80) across all staging items.

**TABLE 7.** Inter-observer agreement analysis (Kappa Coefficient)

Evaluation Project	WB-DWI (κ-value)	PET-CT (κ-value)
**T-Installment Assessment**	0.65	0.78
**N-period judgment**	0.72	0.81
**M-stage determination**	0.80	0.85
**Overall phasing assessment**	0.75	0.82

1 WB-DWI = whole-body diffusion-weighted imaging

1 All Kappa values *p* < 0.001. A Kappa value > 0.75 indicates excellent agreement, while 0.60-0.75 indicates good agreement.

**FIGURE 2.**
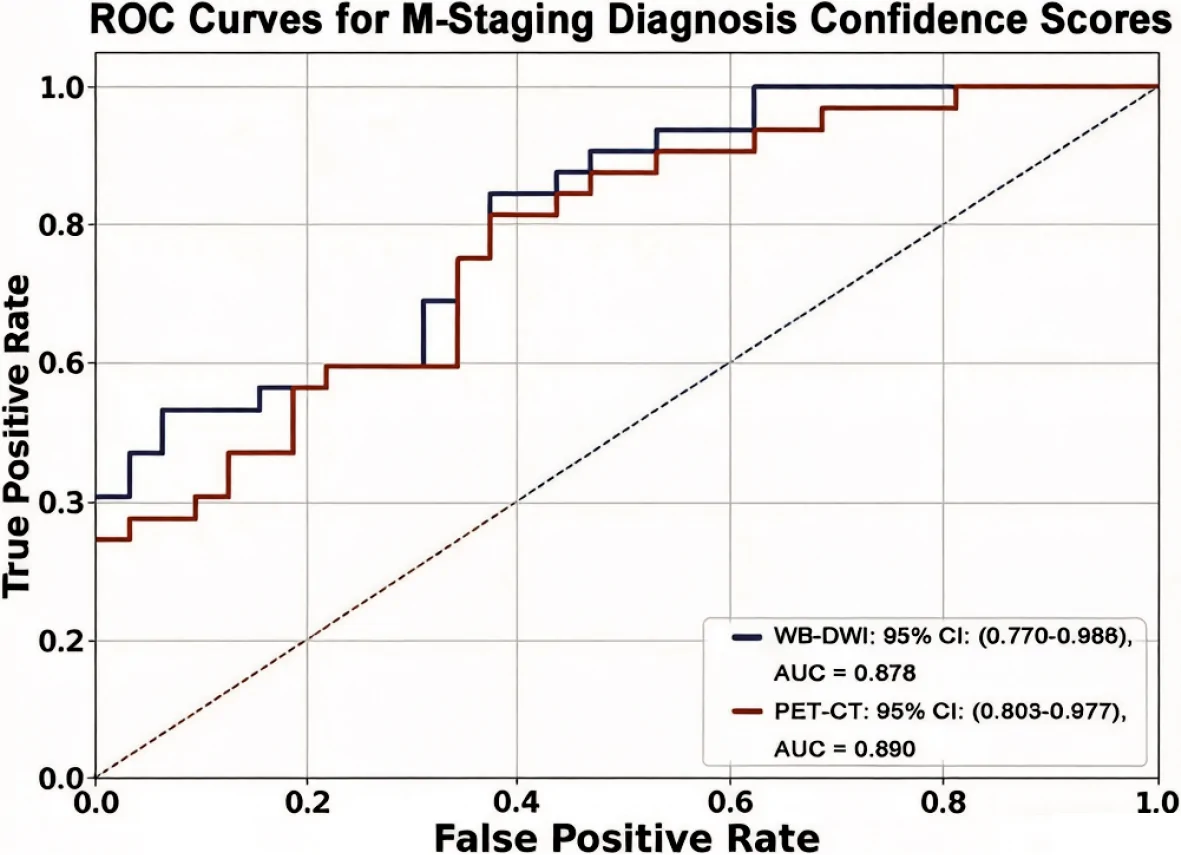
ROC curves for M-staging diagnosis confidence score. AUC = area under the curve; CI = confidence interval; WB-DWI = whole-body diffusion-weighted imaging

Ultimately, the combined diagnostic performance for N staging and M staging was quantified via ROC curve analysis (Table 8, Figure 2). Both techniques demonstrated high accuracy for N staging: WB-DWI (AUC = 0.828, 95% CI: 0.715–0.941) and PET-CT (AUC = 0.890, 95% CI: 0.803–0.977) and M staging WB-DWI (AUC = 0.878, 95% CI: 0.770–0.986), PET-CT (AUC = 0.929, 95% CI: 0.850-1.000) both demonstrated high diagnostic value (AUC > 0.8). DeLong’s test further confirmed that although PET-CT’s AUC values were numerically higher than WB-DWI’s, the diagnostic efficacy (AUC) differences between the two techniques for N staging and M staging were not statistically significant (*p* > 0.05).

**TABLE 8.** ROC curve analysis results (diagnostic performance of N staging and M staging)

Imaging technology	Evaluation project	AUC (95% CI)	Yorden index
**WB-DWI**	N installments	0.828 (0.715–0.941)	0.656
**PET-CT**	N installments	0.890 (0.803–0.977)	0.781
**WB-DWI**	M installments	0.878 (0.770–0.986)	0.756
**PET-CT**	M installments	0.929 (0.850–1.000)	0.859

1 AUC = area under the curve; CI = confidence interval; WB-DWI = whole-body diffusion-weighted imaging

1 The DeLong test revealed no statistically significant difference in AUC between the two techniques for N staging (Z = 1.34, *p* = 0.180) or for M staging (Z = 1.12, *p* = 0.263). # N and M staging diagnoses are based on qualitative assessments and do not utilize a single continuous variable; therefore, numerical cutoff values are not provided.

**TABLE 9.** Detection of small metastatic lymph nodes: comparison of node size between true-positive and false-negative cases on WB-DWI and PET-CT

Imaging modality	Detection outcome	Number of nodes (n)	Short-axis diameter (mm), median (range)	Statistical comparison (TP *vs*. FN)
**WB-DWI**	True-positive	25	8.2 (4.5–18.6)	*p* = 0.008*
	False-negative	7	5.1 (3.2–6.8)	
**PET-CT**	True-positive	29	—(3.8–†)	*p* = 0.278‡
	False-negative	3	4.2 (3.5–5.0)	

1 FN = false negative; TP = True positive: WB-DWI = whole-body diffusion-weighted imaging

* Comparison between TP and FN nodes on WB-DWI using Mann-Whitney U test.

† The upper range of TP node size on PET-CT was not systematically recorded; the smallest detected node was 3.8 mm.

‡ Comparison between TP and FN nodes on PET-CT was not statistically significant (Mann-Whitney U test, *p* = 0.278).w

### Detection of small metastatic lymph nodes by WB-DWI and PET-CT

**FIGURE 3.**
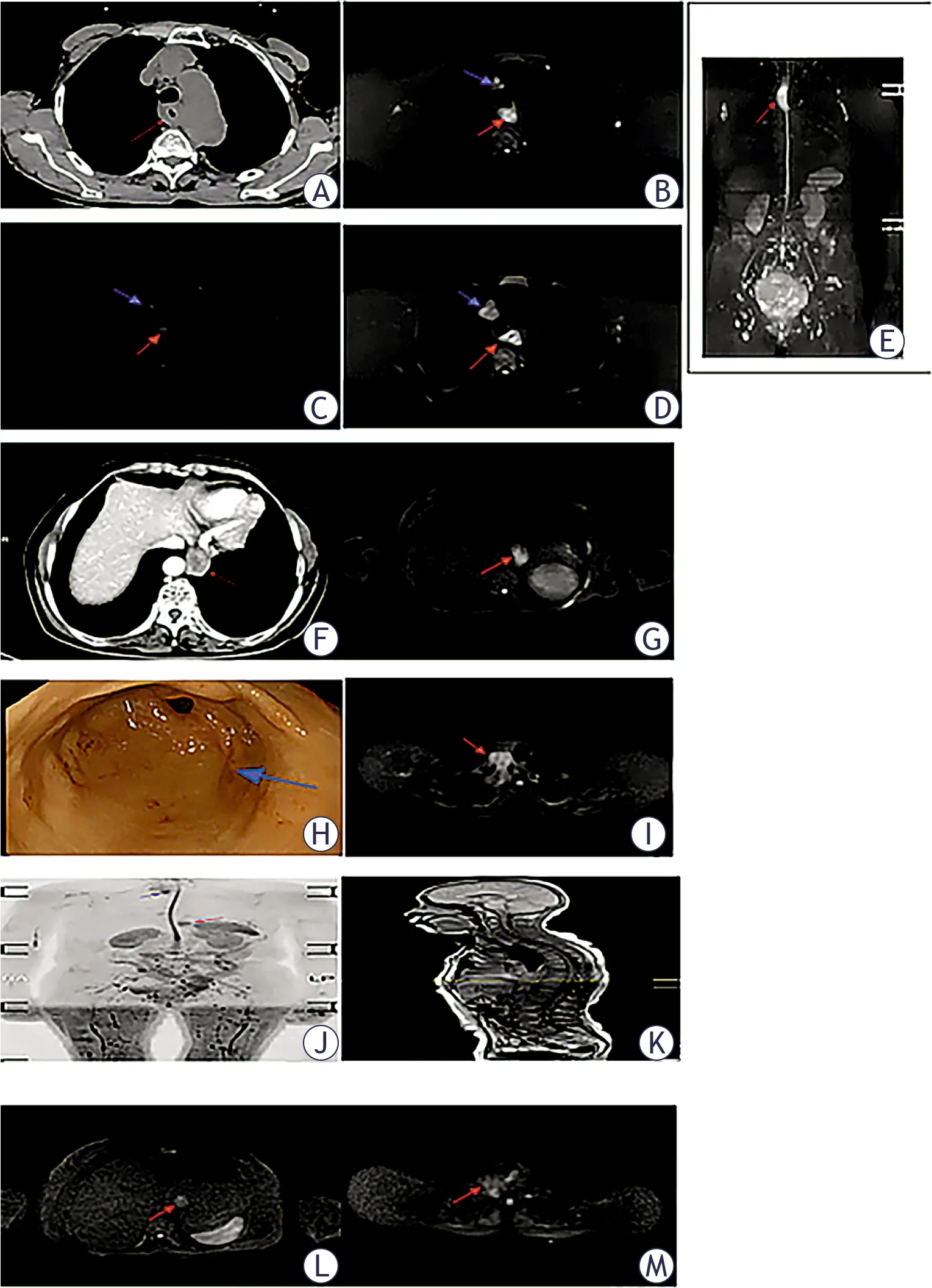
Serial whole-body DWI for dynamic treatment monitoring and restaging in two representative esophageal cancer patients. *Case 1* **(A-E)**: An 86-year-old female with newly diagnosed mid-upper esophageal cancer and mediastinal lymph node metastasis. **(A)** Baseline contrast-enhanced CT shows irregular wall thickening of the esophagus (red arrow) and a small pretracheal lymph node (blue arrow, indeterminate). **(B)** Baseline axial WB-DWI (b = 800 s/mm^2^) demonstrates marked hyperintensity of the primary tumor (red arrow) and the lymph node (blue arrow), indicating metastasis. **(C)** Whole-body DWI maximum intensity projection (MIP) delineates the longitudinal extent of the primary tumor (red arrows), correlating well with barium esophagography and endoscopy. **(D)** After 3 months of chemoradiotherapy, WB-DWI shows reduced signal intensity and size of both lesions, indicating partial response. **(E)** After 6 months, both lesions have progressed (increased size and signal), indicating disease progression. *Case2* **(F-M)**: A 68-year-old female with lower esophageal cancer and right supraclavicular lymph node metastasis. **(F)** Baseline contrast-enhanced CT shows thickening and enhancement of the lower esophagus (red arrow). **(G)** Axial WB-DWI shows a hyperintense primary tumor (red arrow). **(H)** Endoscopy confirms the lower esophageal lesion (blue arrow). **(I)** Axial WB-DWI reveals hyperintense, partially confluent right supraclavicular lymph nodes (blue arrows). **(J)** Whole-body DWI MIP shows the primary tumor (red arrow) and supraclavicular nodes (blue arrows). **(K)** Sagittal WB-DWI localizer image corresponding to **(F)** can guide radiotherapy planning. **(L)** After 8 months of treatment, the primary tumor shows reduced signal and size (red arrow). **(M)** The supraclavicular nodes also show reduced signal (blue arrows), indicating treatment response.

Among the 32 positive lymph node regions (N+) confirmed by pathology or follow-up, the median short diameter of the 25 metastatic lymph nodes detected by WB-DWI was 8.2 mm (range: 4.5–18.6 mm), while the median short diameter of the 7 false-negative lymph nodes missed by WB-DWI was 5.1 mm (range: 3.2–6.8 mm), and the difference was statistically significant (*p* = 0.008). The minimum short diameter of the metastatic lymph nodes detected by WB-DWI was 4.5 mm. In contrast, among the 29 metastatic lymph nodes detected by PET-CT, the minimum short diameter was 3.8 mm, and the median short diameter of the 3 false-negative lymph nodes was 4.2 mm (range: 3.5–5.0 mm). These results indicate that the size of lymph nodes is an important factor affecting the sensitivity of WB-DWI detection, especially for micro-metastatic lymph nodes with a short diameter of < 5 mm, where WB-DWI has a higher risk of missed diagnosis, as shown in Figure 9. The clinical utility of WB-DWI for dynamic treatment monitoring and restaging is illustrated in Figure 3.

Clinical implication: WB-DWI enables radiation-free, longitudinal assessment of tumor burden and treatment response. It can inform dynamic restaging and guide clinical decision-making, particularly when repeated imaging is required.

## Discussion

This study systematically evaluated the diagnostic value of WB-DWI in staging newly diagnosed esophageal cancer through a meticulously designed retrospective comparative analysis, using the widely accepted PET-CT as the reference standard. Our core findings reveal a clinically significant picture: WB-DWI demonstrates diagnostic efficacy comparable to PET-CT for overall TNM staging and detection of distant metastases in esophageal cancer. However, PET-CT retains a certain sensitivity advantage in two critical aspects: precisely determining the local invasion depth of the primary tumor and identifying regional lymph node metastases. This outcome does not simply declare one superior to the other, but provides a nuanced, evidence-based rationale for the differentiated positioning and application of these two imaging modalities in clinical practice. The study’s value lies not only in its comparative conclusions but also in its effective control of baseline confounding factors through propensity score matching. Furthermore, it incorporates multidimensional practical indicators such as image quality, examination duration, and inter-observer consistency, resulting in a more comprehensive and clinically relevant assessment.

Focusing on primary tumor T staging, this study found that PET-CT demonstrated numerically higher accuracy than WB-DWI, although the difference did not reach statistical significance. This trend is closely related to the physical principles of DWI technology and the esophagus’s unique anatomical structure. Accurate T staging relies heavily on clear visualization of the esophageal wall’s layered structures, particularly infiltration of the submucosal and muscular layers.^36^ Although high b-value DWI images can highlight tumor tissue through water diffusion restriction, their relatively low spatial resolution and susceptibility to esophageal artifacts caused by peristalsis, cardiac pulsation, and respiratory motion can obscure fine esophageal wall layers. This may lead to overestimation or underestimation of invasion depth.^37,38^ An earlier study by Penny Fang *et al*. indicated that conventional DWI-MRI achieves approximately 70–80% accuracy in T staging for esophageal cancer, with limitations primarily in distinguishing T2 from T3 disease.^39^ In contrast, the diagnostic CT component of PET-CT, particularly when employing multi-phase contrast-enhanced scanning, provides superior anatomical detail. This facilitates visualization of esophageal wall thickening, rigidity, and blurred relationships with surrounding fat plane critically important for assessing T3/T4 lesions.^40,41^ Our findings align with these technical characteristics. However, it must also be noted that relying solely on the metabolic information from PET-CT has limited value for T staging; its advantage lies more in the fusion interpretation with high-quality CT images. In the future, combining WB-DWI with high-resolution T2-weighted images or even esophagus-specific MRI sequences to construct a multiparametric MRI protocol may be a promising approach to improve the accuracy of local T staging. A similar approach has proven highly successful in the MRI assessment of rectal cancer.

Regarding the diagnosis of regional lymph node metastasis, a critical factor determining treatment strategies – our data demonstrate that PET-CT exhibits significantly higher sensitivity than WB-DWI. This undoubtedly represents one of PET-CT’s core advantages as a gold standard for staging. The fundamental reason is that diagnosing lymph node metastasis relies not only on morphological criteria such as size and shape but also, critically, on its functional metabolic state.^42^ Numerous studies, including those by Sankari Kommi and colleagues, confirm that 18F-FDG PET-CT can detect metabolically active metastatic lymph nodes before they become macroscopically enlarged. This capability alters N staging in approximately 20% of patients with esophageal cancer and influences treatment planning.^43^ Our study observed a PET-CT N staging sensitivity of 90.6%, consistent with findings from multiple prior meta-analyses.^44^ While WB-DWI can also suggest metastasis through restricted diffusion of water molecules within lymph nodes (high signal), the specificity of its signal changes remains challenging.^14^ Benign lymph node lesions, such as reactive hyperplasia and inflammation, may also cause diffusion restriction, whereas certain micrometastases or lymph nodes with low cell density may not exhibit the typical high signal.^45^ In this study, the sensitivity of WB-DWI in N staging was lower than that of PET-CT (78.1% *vs*. 90.6%). Part of the reason can be attributed to the limited detection ability of DWI for small-volume metastatic lymph nodes. Our data indicate that the minimum short diameter of metastatic lymph nodes detected by WB-DWI is 4.5 mm, and for micro-metastatic foci with a short diameter of <5 mm, the missed diagnosis rate significantly increases. This finding is consistent with previous studies: the spatial resolution of DWI (usually 1.5–2.0 mm within-layer resolution) and partial volume effect make it difficult to distinguish the signal of lesions smaller than 3–4 mm from background noise.^46^ In contrast, PET-CT can detect lymph nodes with a short diameter of only 3–4 mm and active metabolism, thus having a greater advantage in the detection of early micrometastases. Clinically, for small lymph nodes that are highly suspected but negative in WB-DWI, it is recommended to further evaluate by combining endoscopic ultrasound or PET-CT. Notably, both techniques demonstrated identical specificity in this study, though neither achieved perfection. This suggests that false positives remain an inherent issue across all functional imaging modalities. In clinical practice, suspected lymph nodes identified by PET-CT or WB-DWI, particularly in critical regions, may still require confirmation via endoscopic ultrasound-guided fine-needle aspiration biopsy. This raises a deeper consideration: For N staging, future imaging advancements should not solely pursue higher sensitivity. Equally important may be enhancing specificity through quantitative parameters, such as ADC values, SUVmax ratios, or texture features to reduce unnecessary invasive procedures.

The most compelling and noteworthy finding in this study is the comparable performance of WB-DWI versus PET-CT in detecting distant metastases. No statistically significant differences were observed between the two modalities in sensitivity, specificity, or AUC. This result has significant implications for advancing the clinical application of WB-DWI. The presence of distant metastasis directly determines disease staging and the feasibility of curative treatment goals. WB-DWI inherently offers the advantage of performing whole-body screening in a single examination.^46^ Our findings are supported by a series of recent studies. For instance, in a comparative study across multiple malignancies, Mahmoud Rezk *et al*. found that DWI-based breast imaging (DWIBS) technology demonstrated comparable capability to PET-CT in detecting distant metastases.^47^ Specifically for esophageal cancer, some scholars have explored the value of WB-DWI in detecting liver, bone, and non-regional lymph node metastases. Particularly for bone metastases, studies indicate that DWI is highly sensitive to abnormal signals from early intramedullary lesions, sometimes even outperforming bone scans and CT, and effectively complements PET-CT’s metabolic detection capabilities.^48,49^ The M staging gold standard in this study combined pathology and long-term follow-up, thereby enhancing the robustness of the conclusions. From a pathophysiological perspective, metastatic lesions typically exhibit high cellular density and active proliferation, manifesting on DWI as markedly restricted diffusion with high contrast, making them readily identifiable. Although PET-CT may hold an advantage over WB-DWI in detecting certain specific metastases (*e.g*., pulmonary micronodules) due to CT’s superior spatial resolution, WB-DWI’s radiation-free nature and absence of radioactive tracer injection make it particularly suitable for patients requiring multiple follow-up examinations, younger patients, or those with contrast/tracer allergies.^50^ This advantage is significant, directly impacting patient safety over the long term and the repeatability of examinations.

Beyond its diagnostic performance in initial staging, WB-DWI demonstrates significant value in longitudinal disease assessment. As shown in Figure 3, serial WB-DWI examinations can visually capture tumor burden changes, from baseline through partial response to progression in a radiation-free manner. This capability allows clinicians to dynamically revise the clinical stage based on treatment response, which is particularly important for patients receiving neoadjuvant therapy or palliative treatment where repeated imaging is required. In contrast, repeated PET-CT is often limited by cumulative radiation exposure and higher costs. Thus, WB-DWI may serve as a practical alternative for treatment monitoring and restaging, especially in resource-limited settings or for patients requiring frequent follow-up.

Beyond the core diagnostic metrics mentioned above, this study’s evaluation of image quality, examination efficiency, and observer consistency provides indispensable practical information for clinical decision-making. Objective measurements and subjective ratings of image SNR consistently demonstrate superior image quality in PET-CT, primarily due to its technical maturity and higher spatial resolution.^51^ However, a critical balancing factor is examination duration. This study clearly documented that WB-DWI examination times were significantly shorter than PET-CT. In a high-efficiency radiology department, shorter examination times translate to increased patient throughput and reduced appointment backlogs. Simultaneously, shorter examination times enhance patient comfort and cooperation, particularly for advanced cancer patients in poor physical condition. Inter-observer agreement analysis revealed both techniques achieved good to excellent consistency, though PET-CT generally yielded slightly higher Kappa values. This suggests PET-CT interpretation may hold greater potential for standardization due to its distinct imaging features (*e.g*., hypermetabolic lesions) and accumulated diagnostic expertise. Nevertheless, WB-DWI achieved Kappa values at the “good” level or higher, indicating that radiologists can reliably interpret WB-DWI images with appropriate training, laying the groundwork for its clinical adoption. These “non-diagnostic” yet critical factors collectively paint a more complete picture: PET-CT offers more “refined” diagnostic information in certain aspects, while WB-DWI provides a faster, radiation-free alternative or supplement with reliable diagnostic efficacy, particularly for wholebody screening.

Placing this study within a broader academic context reveals its innovation and significance across multiple dimensions. Methodologically, propensity score matching to address selection bias inherent in retrospective studies provides a more equitable basis for comparing the WB-DWI and PET-CT groups. This approach, not widely adopted in previous comparative studies, enhances the credibility of the conclusions. In terms of research content, we transcended the traditional paradigm of solely comparing diagnostic sensitivity and specificity. By incorporating metrics directly impacting clinical adoption and workflow, such as image quality, examination time, and observer consistency into our systematic evaluation, the study conclusions offer more direct reference value for hospital administrators and clinicians. Clinically, this study provides evidence for individualized imaging pathways in esophageal cancer. For patients seeking a cure and requiring precise N staging to determine the necessity of neoadjuvant therapy, PET-CT may remain the preferred choice. However, for staging evaluation, treatment follow-up, and especially for patient groups needing to avoid radiation exposure, WB-DWI is undoubtedly an attractive alternative. Furthermore, considering the high cost and limited availability of PET-CT equipment, WB-DWI emerges as a more accessible MRI technique, highlighting its value in resource-constrained regions.

Looking ahead, several valuable research directions can be extended based on the findings of this study. One is to explore the correlation between WB-DWI quantitative parameters, such as lesion ADC values and their histogram characteristics and tumor pathological grading, treatment response, and prognosis. Preliminary studies exist, such as Qingxue Cong *et al.’s* investigation into the relationship between esophageal cancer ADC values and tumor invasiveness, which will help advance WB-DWI from morphofunctional diagnosis to higher-level applications, such as prognosis prediction and treatment efficacy monitoring.^52^ Second, investigating the value of integrating or combining WB-DWI with PET-CT. Rather than choosing one over the other, this considers whether their information complements each other in complex cases, achieving a “1+1>2” diagnostic effect. For instance, combining PET’s high metabolic specificity with DWI’s high soft tissue contrast may further enhance diagnostic confidence for peritoneal and small lymph node metastases. Third, with the explosive growth of artificial intelligence, particularly deep learning, in medical image analysis, developing automated segmentation, feature extraction, and staging prediction models based on WB-DWI or PET-CT images holds promise. This could partially address interobserver variability and uncover deep imaging biomarkers invisible to the human eye, potentially representing a next-generation technological breakthrough for more precise, objective staging.

Of course, this study also has certain limitations that should be considered when interpreting the results. First, despite the use of propensity score matching (PSM), the retrospective study design itself cannot completely eliminate all potential biases. Second, although the sample size was estimated and met statistical requirements, a larger multicenter sample would provide more robust estimates and enable more meaningful subgroup analyses. As Yousafzai *et al*. noted in their discussion of emerging cancer technologies, standardization of analytical methods and rigorous external validation are critical steps for any diagnostic technology to advance toward routine clinical application.^53^ Third, the gold standard pathological TNM staging was not available for all patients. For non-surgical patients, we relied on comprehensive clinical follow-up diagnoses. Despite extended follow-up periods and strict criteria, this approach inherently differs from pathological standards. Fourth, the WB-DWI technical parameters and interpretation experience in this study are based on a single center. Extending these conclusions to other institutions using different MRI equipment, sequences, and parameters may require further validation. Fifth, the accuracy of T staging in this study was derived based on the examination protocol that included diagnostic enhanced CT. This result may not be applicable to the routine PET/CT examinations where only low-dose non-contrast CT is used. For units where diagnostic CT cannot be performed due to equipment limitations, the value of PET/CT for T staging will be significantly reduced. In such cases, it is recommended that patients undergo separate enhanced CT or MRI examinations. Sixth, in this study, the accuracy analysis of T staging only included patients who underwent surgery (accounting for 75.0% of the matched cohort). This may introduce selection bias, as surgical patients are usually staged relatively early or are potentially resectable. For patients with advanced disease who did not undergo surgery, the T staging cannot obtain the pathological gold standard. Therefore, the conclusions of this study have limited value for the assessment of T staging in locally advanced and unresectable esophageal cancer. Seventh, due to the retrospective design and incompatible DICOM archiving formats between PET-CT and WB-DWI workstations, we were unable to retrieve directly comparable PET-CT images for the same patients at identical time points. Therefore, Figure 3 presents only WB-DWI serial images to demonstrate its clinical utility in treatment monitoring, but side-by-side PET-CT/WB-DWI comparisons are not provided. Future prospective studies should include paired imaging to enable direct visual comparison.

## Conclusions

In summary, this study rigorously demonstrated, through comparative analysis, that WB-DWI is an effective tool for staging esophageal cancer. It exhibits comparable efficacy to PET-CT in overall staging and detection of distant metastases, while offering distinct advantages of radiation-free imaging and a shorter examination duration. Although slightly less sensitive than PET-CT for local T staging and regional lymph node staging, it performs well overall and is well-suited for its critical application in specific clinical scenarios. The findings not only enrich the evidence base for esophageal cancer imaging assessment but also provide practical guidance for clinicians to individualize the selection of optimal imaging protocols based on patient-specific conditions, healthcare resources, and technical capabilities. Advances in imaging technology ultimately serve more precise diagnosis and treatment, and WB-DWI, with its unique value, is securing a solid position within the multidisciplinary diagnostic and therapeutic landscape of esophageal cancer.
